# Comparative studies on the genetic, antigenic and pathogenic characteristics of Bokeloh bat lyssavirus

**DOI:** 10.1099/vir.0.065953-0

**Published:** 2014-08

**Authors:** Tobias Nolden, Ashley C. Banyard, Stefan Finke, Anthony R. Fooks, Dennis Hanke, Dirk Höper, Daniel L. Horton, Thomas C. Mettenleiter, Thomas Müller, Jens P. Teifke, Conrad M. Freuling

**Affiliations:** 1 Friedrich-Loeffler-Institut (FLI), Federal Research Institute for Animal Health, Institute of Molecular Virology and Cell Biology, WHO Collaborating Centre for Rabies Surveillance and Research, Südufer 10, D-17493 Greifswald – Insel Riems, Germany; 2 Animal Health and Veterinary Laboratories Agency – Weybridge, Wildlife Zoonoses and Vector-borne Diseases Research Group, Woodham Lane, Surrey KT15 3NB, UK; 3 University of Liverpool, Department of Clinical Infection, Microbiology and Immunology, Liverpool L3 5TQ, UK; 4 FLI, Institute of Diagnostic Virology, WHO Collaborating Centre for Rabies Surveillance and Research, Südufer 10, D-17493 Greifswald–Insel Riems, Germany; 5 School of Veterinary Medicine, University of Surrey, Guildford, UK; 6 FLI, Department of Animal Husbandry and Biorisk Management, WHO Collaborating Centre for Rabies Surveillance and Research, Südufer 10, D-17493 Greifswald – Insel Riems, Germany

## Abstract

Bokeloh bat lyssavirus (BBLV), a novel lyssavirus, was isolated from a Natterer’s bat (*Myotis nattererii*), a chiropteran species with a widespread and abundant distribution across Europe. As a novel lyssavirus, the risks of BBLV to animal and human health are unknown and as such characterization both *in vitro* and *in vivo* was required to assess pathogenicity and vaccine protection. Full genome sequence analysis and antigenic cartography demonstrated that the German BBLV isolates are most closely related to European bat lyssavirus type 2 (EBLV-2) and Khujand virus and can be characterized within phylogroup I. *In vivo* characterization demonstrated that BBLV was pathogenic in mice when inoculated peripherally causing clinical signs typical for rabies encephalitis, with higher pathogenicity observed in juvenile mice. A limited vaccination-challenge experiment in mice was conducted and suggested that current vaccines would afford some protection against BBLV although further studies are warranted to determine a serological cut-off for protection.

Rabies is one of the most feared viral zoonotic diseases. It is caused by viruses classified within the *Lyssavirus* genus, family *Rhabdoviridae*, order *Mononegavirales* (Kuzmin & Tordo, 2012). The lyssavirus negative-sense single stranded RNA genome encodes five viral proteins: the nucleoprotein (N), phosphoprotein (P), matrix protein (M), glycoprotein (G) and the viral RNA polymerase (L) (Conzelmann, 1998). The *Lyssavirus* genus comprises several virus species (Dietzgen *et al.*, 2012; World Health Organization, 2013), of which the majority are associated with bats. In Europe, between 1977 and 2012 approximately one thousand bat rabies cases were reported (Schatz *et al.*, 2013). The majority of these cases were characterized as European bat lyssavirus type 1 (EBLV-1), predominantly isolated from serotine bats (*Eptesicus serotinus*, *E. isabellinus*) (Banyard *et al.*, 2011). Alongside EBLV-1 infections, about 24 cases of EBLV-2 have been reported from various European countries.

The discovery of novel lyssaviruses is usually limited to the detection of a single isolate within a bat species (Banyard *et al.*, 2011). Within a short time period, Bokeloh bat lyssavirus (BBLV) has been isolated from European Natterer’s bats (*Myotis nattererii*) on three separate occasions. This apparent emergence of the BBLV in Natterer’s bat populations in Germany (Freuling *et al.*, 2011) and France (Picard-Meyer *et al.*, 2013) has caused some concern and a closer characterization of the viruses involved was necessary to evaluate the potential risks. We therefore took two BBLV isolates from Germany and investigated their properties *in vitro* and *in vivo*.

Full genome sequences of the two German BBLVs were obtained using next generation sequencing (NGS) technology (454 Genome Sequencer FLX platform, Roche) (Freuling *et al.*, 2013). Other full-length lyssavirus genome sequences were obtained from the NCBI GenBank, aligned and concatenated using mega5 (Tamura *et al.*, 2011). The antigenic relationships between BBLV and a global panel of lyssaviruses were quantified and visualized, using antigenic cartography techniques described previously (Horton *et al.*, 2010; Smith *et al.*, 2004), whereby the ability of a panel of hyperimmune rabbit sera to neutralize a fixed quantity of each virus was measured. Pathogenicity in mice was studied for the two BBLV isolates from Germany. Additionally, representatives of rabies virus (RABV), EBLV-1 and EBLV-2 were used for comparison.

Three-week- and 8-week-old BALB/cs mice were used throughout the study (FLI stock). Mice were provided with food and water *ad libitum.* Groups of mice (*n* = 5–7) were challenged by (i) intramuscular (IM) inoculation with two different dosages, i.e. low-dose (1×10^2^ TCID_50_ per 30 µl) and high-dose (1×10^5^ TCID_50_ per 30 µl) and (ii) direct intracranial (IC) inoculation of a low dose of virus (1×10^2^ TCID_50_ per 30 µl). Animals found with scores of three or above were humanely euthanized.

In parallel experimental studies, groups of five genetically homogeneous 5-week-old female Swiss OF1 mice (Charles River, France) were challenged with high doses (10^5.4^ TCID_50_ per 30 µl) and medium doses (10-fold dilution) of BBLV (LabNo. 21961, GenBank JF311903) by IC and IM inoculation. Brains were divided at post-mortem and assessed for the presence of virus antigen using the fluorescent antibody test (FAT) (Dean *et al.*, 1996). The remaining brain section was fixed in 10 % buffered formalin, embedded in paraffin wax, sectioned and stained with haematoxylin and eosin. Immunohistochemistry was performed as described previously (Schatz *et al.*, 2014) using a polyclonal rabbit serum N161-5 (Orbanz & Finke, 2010). Individual serum samples taken post-mortem were tested for the presence of virus neutralizing antibodies (VNA) using a modified rapid fluorescent focus inhibition test (RFFIT) as described previously (Vos *et al.*, 2004) with BBLV as test virus. For conversion into international units, a heterologous WHO international standard immunoglobulin (2nd human rabies immunoglobulin preparation, National Institute for Standards and Control, Potters Bar, UK) adjusted to 2.5 IU ml^−1^ for BBLV was used. For vaccination–challenge experimentation, mice were vaccinated via the intraperitoneal route with 500 µl of a commercially available human rabies vaccine (VERORab). At 21 days post-vaccination, mice were tail bled and sera were assessed for the presence of neutralizing antibodies using a pseudotype neutralization assay as described by Wright *et al.* (2009). Once titres had been assessed, mice were grouped and challenged by the IC route with either neat or a 10^−1^ dilution of BBLV. Animals were then monitored for 28 days post-challenge and the development of clinical disease assessed. All *in vivo* work was undertaken in compliance with Home Office licence guidance (PPL 70/7934) or was approved by the responsible authorities (AZ LALLF M-V/TSD/7221.3-2.1-002/11).

Sequence analysis using concatenated N-P-M-G-L gene sequences demonstrated 80 % and 79 % similarity between all BBLV sequences and the sequence of the most similar viruses, Khujand virus (KHUV) and EBLV-2, respectively, while the intra-species similarity values ranged between 92.7 % and 98.6 %. Phylogenetic analysis, performed on the ectodomain of the G-gene, demonstrated a limited relatedness between BBLV and other lyssavirus species (Fig. 1a). Antigenic relationships based on cartography largely reflected the genetic relationships, with BBLV being distinguishable from all characterized lyssaviruses, but closely related to phylogroup I viruses. With an average of 3.9 and 4.3 antigenic units (AU), BBLV is antigenically equidistant from RABV and EBLV-2, but further from EBLV-1 viruses (average 6.5 AU; Fig. 1b).

**Fig. 1.**
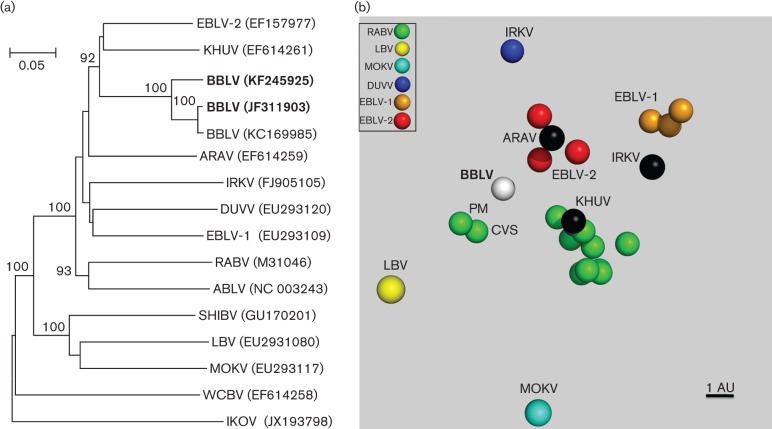
(a) Phylogenetic relationship of BBLV and other representative lyssaviruses inferred from the glycoprotein ectodomain gene sequences using mega5 software (neighbour-joining method, p-distance model, 1000 replicates). Bar, 0.05 substitutions per nucleotide position. (b) Antigenic map showing the antigenic relationship between BBLV and other lyssaviruses. Viruses (spheres) are positioned such that the distance from each serum to each virus is determined by the neutralization titre. Multidimensional scaling is used to position both sera and viruses relative to each other, so orientation of the map within the axes is free. Bar shows approximately one antigenic unit (AU), equivalent to a twofold dilution in antibody titre. Sera have been removed for clarity (viewed using Pymol, DeLano Scientific). Raw data are provided in Table S1 (available in the online Supplementary Material).

Generally, no differences in the development of clinical disease were observed between the different viruses used for infection. When the pathogenicity of the original BBLV JF311903 was compared with that of EBLV-1 and -2 as well as the cell-culture-adapted pathogenic CVS-11 strain and the tissue-culture-attenuated SAD L16 strain, mice challenged IM with BBLV died between days 10 and 12 (1×10^5^ TCID_50_ per 30 µl; Fig. 2a) post-infection (p.i.), or at 13 (1×10^2^ TCID_50_ per 30 µl; not shown) days p.i. For EBLV-1, all mice in both high and low-dose groups developed clinical disease and were euthanized by 12 and 15 days p.i., respectively. While all mice developed clinical disease following IC inoculation, following peripheral inoculation more than half of the mice (*n* = 3, 60 %) survived a low dose of CVS-11 (not shown) and 20 % survival was seen following EBLV-2 inoculation irrespective of dose. SAD L16 in comparison showed 100 % survival when a low dose was inoculated peripherally (1×10^2^ TCID_50_ per 30 µl; not shown) but residual pathogenicity was seen when the high dose of 1×10^5^ TCID_50_ per 30 µl was administered via the IM route (Fig. 2a). Similar to the other lyssaviruses investigated, there was mild lympho-plasmocytic encephalitis following BBLV infection, and BBLV antigen was detected by immunohistochemistry in various areas of brain (Fig. 2b, VII, VIII, IX). Interestingly, in comparison with EBLV-1, the accumulation of viral antigen in the neuronal cytoplasm was less pronounced in BBLV infected animals. However, tropism of the virus for neurons and glial cells appeared unaffected (Fig. 2b).

**Fig. 2.**
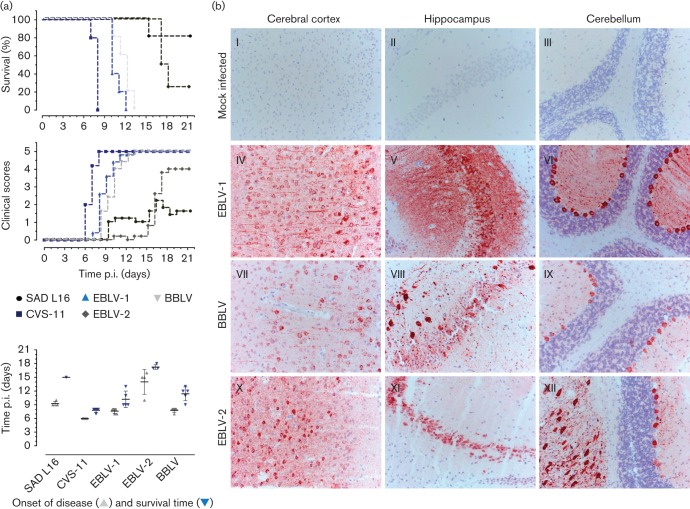
Pathogenicity of BBLV compared to SAD-L16 and CVS-11 and EBLV-1 and -2. Three-week-old BALB/c mice were inoculated via the IM and IC routes of infection. Clinical signs of rabies were recorded daily. (a) Kaplan–Meyer plots, clinical scores as well as onset of disease and survival times were shown for the IM-infected animals (1×10^5^ TCID_50_, *n* = 5). (b) Immunohistochemical detection of viral N antigen in the cortex (first column), hippocampus (second column) and cerebellum (third column). BALB/c mice (1×10^5^ TCID_50_; IM-inoculated) with clinical scores ≥3 were euthanized and dissected brains were formalin fixed and paraffin wax embedded (FFPE). Immunostaining of the FFPE sections with N-protein-specific rabbit serum (red signal) showed numerous neurons with axons and dendrites containing characteristic Negri bodies in EBLV-1 (IV, V, VI); BBLV (VII, VIII, IX) and EBLV-2 (X, XI, XII) infected brains. Mock-infected control brains (I, II, III) did not show any specific signal. Haematoxylin counterstain (blue).

Parallel experimental infections with BBLV JF311903 in a separate laboratory using 4-week-old OF1 mice confirmed these results, although there were slight differences in onset of disease. The incubation period in OF1 mice inoculated IC was similar to that seen in 3- to 4-week-old BALB/c mice at 5–6 days. The incubation period and survival in OF1 mice inoculated IM were shorter than in the BALB/c mice at 7–10 days with no apparent dose effect in either the incubation period or survival (20–40 %).

In a follow-up experiment comparing the two BBLV isolates JF311903 and KF245925, all 3- to 4-week-old BALB/c mice inoculated IC developed clinical disease consistent with lyssavirus infection by 5–6 days p.i. (Fig. 3a, c) and all animals inoculated by this route succumbed by 9 days p.i. (Fig. 3d). There were no significant differences in onset of disease, mean survival time, body mass reduction or the development of rabies clinical signs between the two isolates (Table S2, Fig. 3a, c, d).

**Fig. 3.**
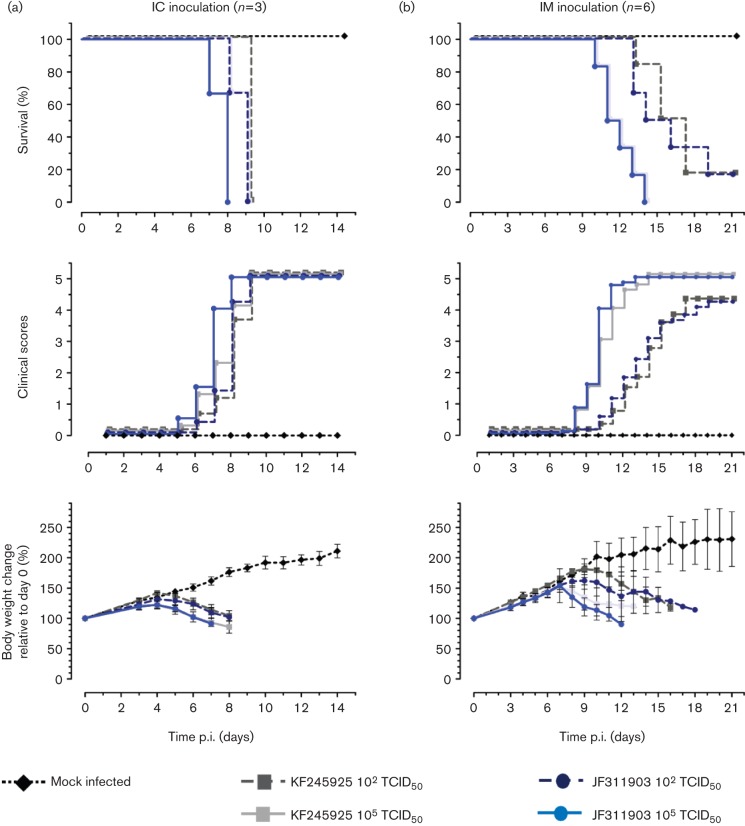

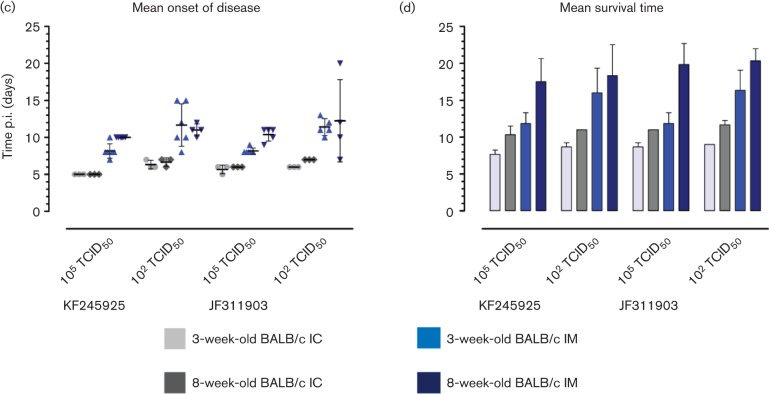
Pathogenicity of BBLV isolates JF311903 and KF245925 in 3- and 8-week-old BALB/c mice. Mice were inoculated with 1×10^2^ and 1×10^5^ TCID_50_ via the intracranial (a) or via the intramuscular (b) route of infection. Mice were monitored daily for clinical signs of rabies using a scoring system: 0 (no effects), 1 (ruffled fur, hunched back), 2 (slow or circular movements, gait affected), 3 (trembling, shaking and lameness) and 4 (paralysis). Mean onset of disease (c) and survival time (d) were calculated from clinical data with n = 3 for IC and n = 6 for IM inoculated mice.

Both BBLV isolates, JF311903 and KF245925, were less pathogenic in IM-inoculated 8- to 9-week-old BALB/c mice (Table S2) and the time until the onset of disease and mean survival time of diseased mice were prolonged (Fig. 3c, d). In 3- to 4-week-old mice, a statistically significant difference (*P*<0.05; log-rank Mantel-Cox test) in survival was observed between the high and low-dose challenge groups, i.e. all mice challenged IM with the high dose succumbed to disease between 11 and 14 days p.i. (mean 11.8±1.2 days) while five out of six mice challenged with the low dose were euthanized with clinical disease between 13 and 17 days p.i. (mean 16.0±1.2 days; Fig. 3b).

From a serological perspective, 47 % of the mice (*n* = 9/19) that survived BBLV infection had detectable levels of VNAs ranging between 1.8 and 10.2 IU ml^−1^. Only one of those was a 3-week-old mouse, the rest were among the 8-week-old mice. Here, with one exception, all serologically positive mice were from the high-dose IM group.

Using a reduced, modified version of the standard NIH vaccine efficacy test, vaccination and challenge with a high dose of BBLV, administered via the IC route, was performed (Horton *et al.*, 2014). Post-vaccinal VNAs varied between 0.03 and 32 IU ml^−1^ with only one mouse failing to seroconvert (>0.5 IU ml^−1^); 60 % (*n* = 12) developed neutralizing titres of between 4 and 8 IU ml^−1^ and the remaining 30 % (*n* = 6) had titres of >10 IU ml^−1^. Following assessment of neutralizing titres, mice were grouped and challenged with either 10^5.4^ TCID_50_ per 30 µl of BBLV or a 10-fold dilution of this dose. All but one mice that had neutralizing titres of >10 IU ml^−1^ survived challenge whilst all other animals succumbed to infection after challenge with BBLV via the IC route. All unvaccinated control animals succumbed within 5 days of challenge.

Bats infected with lyssaviruses pose a low, but undeniable, threat to both veterinary and human health. In fact, across Europe, both EBLV-1 and EBLV-2 have caused a total of five human casualties (Johnson *et al.*, 2010), and spill-overs of EBLV-1 have been detected in a stone marten (*Martes foina*) (Müller *et al.*, 2004), sheep (Tjørnehøj *et al.*, 2006) and two cats (Dacheux *et al.*, 2009). Since the Natterer’s bat is an abundant bat species in Europe, a balanced assessment of risk to human and animal health for this novel lyssavirus is needed.

As expected from phylogenetic assessment (Fig. 1a) and typing with monoclonal antibodies (Freuling *et al.*, 2011), BBLV grouped within phylogroup I, but, in contrast to the observed genetic relationships, was antigenically equidistant to RABV and EBLV-2, but not to KHUV (Fig. 1b). It was previously suggested for other lyssaviruses that genetic differences in the G-ectodomain would account for differences in antibody titre against a virus (Horton *et al.*, 2010); however, the reason why KHUV is not equally neutralized is not clear.

Pathogenicity studies demonstrated that BBLV is pathogenic when inoculated by both the IC and IM routes and can cause rabies (Fig. 2). Regardless of the close genetic relatedness of BBLV to EBLV-2, BBLV appears to be as pathogenic as EBLV-1 in mice. The BBLV isolates assessed did not differ in their potential to induce rabies in the mouse model, and thus sequence differences observed between these isolates (Fig. 1a) had no detectable influence on pathogenicity (Fig. 3, Table S2). Given the close genetic relatedness of JF311903 with the French isolate (GenBank KC169985; Fig. 1a), a similar pathogenicity can be assumed. Here, the onset of disease following BBLV infection and mean survival times depended on inoculation dose when administered peripherally, albeit to a lesser extent than observed for the other viruses investigated, and survival rates strongly depended on the age of infected mice. One possible explanation for the apparent absence of seroconversion in low-dose IM-infected 8-week-old mice is that the dose was insufficient to produce local virus replication in the muscle necessary to induce a measurable humoral immune response.

The limited vaccination-challenge study performed suggested that mice with high neutralizing titres were able, in the main, to survive challenge with a high dose of BBLV administered via the IC route. These results concur with those of previous studies of RABV and EBLV, where vaccination showed a similar lack of protection to IC challenge with wild-type RABV and EBLV-1, whilst protection was afforded against EBLV-2 (Brookes *et al.*, 2005). Furthermore, *in vitro* studies have suggested that for protection against the non-rabies lyssaviruses, titres post-vaccination need to be greater than required to efficiently neutralize classical rabies virus strains (Brookes *et al.*, 2005). Certainly, from the present study, the response to post-vaccinal challenge highlights the need to further evaluate the ability of current rabies vaccines to protect against BBLV infection.
